# Schmallenberg Virus Circulation in *Culicoides* in Belgium in 2012: Field Validation of a Real Time RT-PCR Approach to Assess Virus Replication and Dissemination in Midges

**DOI:** 10.1371/journal.pone.0087005

**Published:** 2014-01-23

**Authors:** Nick De Regge, Maxime Madder, Isra Deblauwe, Bertrand Losson, Christiane Fassotte, Julie Demeulemeester, François Smeets, Marie Tomme, Ann Brigitte Cay

**Affiliations:** 1 Operational Direction Viral Diseases, CODA-CERVA, Brussel, Belgium; 2 Department of Biomedical Sciences, Institute of Tropical Medicine, Antwerp, Belgium; 3 Department of Infectious and Parasitic Diseases, University of Liège, Liège, Belgium; 4 Life Science Department, Walloon Agricultural Research Center (CRA-W), Gembloux, Belgium; 5 Department of Veterinary Tropical Diseases, University of Pretoria, Pretoria, South Africa; Thomas Jefferson University, United States of America

## Abstract

Indigenous *Culicoides* biting midges are suggested to be putative vectors for the recently emerged Schmallenberg virus (SBV) based on SBV RNA detection in field-caught midges. Furthermore, SBV replication and dissemination has been evidenced in *C. sonorensis* under laboratory conditions. After SBV had been detected in *Culicoides* biting midges from Belgium in August 2011, it spread all over the country by the end of 2011, as evidenced by very high between-herd seroprevalence rates in sheep and cattle. This study investigated if a renewed SBV circulation in midges occurred in 2012 in the context of high seroprevalence in the animal host population and evaluated if a recently proposed realtime RT-PCR approach that is meant to allow assessing the vector competence of *Culicoides* for SBV and bluetongue virus under laboratory conditions was applicable to field-caught midges. Therefore midges caught with 12 OVI traps in four different regions in Belgium between May and November 2012, were morphologically identified, age graded, pooled and tested for the presence of SBV RNA by realtime RT-PCR. The results demonstrate that although no SBV could be detected in nulliparous midges caught in May 2012, a renewed but short lived circulation of SBV in parous midges belonging to the subgenus *Avaritia* occured in August 2012 at all four regions. The infection prevalence reached up to 2.86% in the south of Belgium, the region where a lower seroprevalence was found at the end of 2011 than in the rest of the country. Furthermore, a frequency analysis of the Ct values obtained for 31 SBV-S segment positive pools of *Avaritia* midges showed a clear bimodal distribution with peaks of Ct values between 21–24 and 33–36. This closely resembles the laboratory results obtained for SBV infection of *C. sonorensis* and implicates indigenous midges belonging to the subgenus *Avaritia* as competent vectors for SBV.

## Introduction

Schmallenberg virus is an orthobunyavirus belonging to the Simbu serogroup and was first identified in 2011 [1]. It causes a non-specific syndrome including high fever, decrease in milk production and severe diarrhoea in adult cattle [1] and is furthermore responsible for abortions, stillbirths and congenital malformations such as the hydranencephaly-arthrogryposis syndrome in cattle, sheep and goat [2]–[4]. Since its initial appearance in Germany and The Netherlands, followed by Belgium, the United Kingdom and France, SBV has now spread over much of Europe and beyond [5]–[8].

*Culicoides* biting midges have in the meantime been implicated as putative vectors of SBV what is in line with the knowledge that related viruses belonging to the same serogroup like Akabane and Aino virus are transmitted by midges and mosquitoes [9]–[12]. Several studies have detected SBV in (heads of) field caught Obsoletus complex, *C. dewulfi, C. chiopterus* and *C. punctatus* midges [13]–[19] and SBV replication and dissemination in *C. sonorensis* midges has been shown under laboratory conditions [20].

In Belgium, SBV had spread all over the country by the end of the vector season of 2011 as evidenced by a very high between-herd seroprevalence in sheep and goats [21], [22]. In the southern part of the country, a relatively lower within-herd seroprevalence was found. This was in line with the observation that SBV positive *Culicoides* could be found at different sampling regions in the country, except in the most southern trapping locations [13] (unpublished results).

Despite the overall presence of anti-SBV antibodies in the host populations [21]–[23] which have been shown to protect against challenge infections under experimental conditions [24], [25], clear indications of a renewed SBV circulation in 2012 in sheep and cattle in Belgium and surrounding countries have been found [26]–[29]. In this study, the presence of SBV in field trapped *Culicoides* at different locations in Belgium in 2012 was examined and compared with the results from midges in 2011. Furthermore the results were used to evaluate a recently proposed method to assess vector competence of *Culicoides* for arboviruses via semi-quantitative RT-PCR [20], [30].

## Materials and Methods

### *Culicoides* Trapping

**Figure 1:**
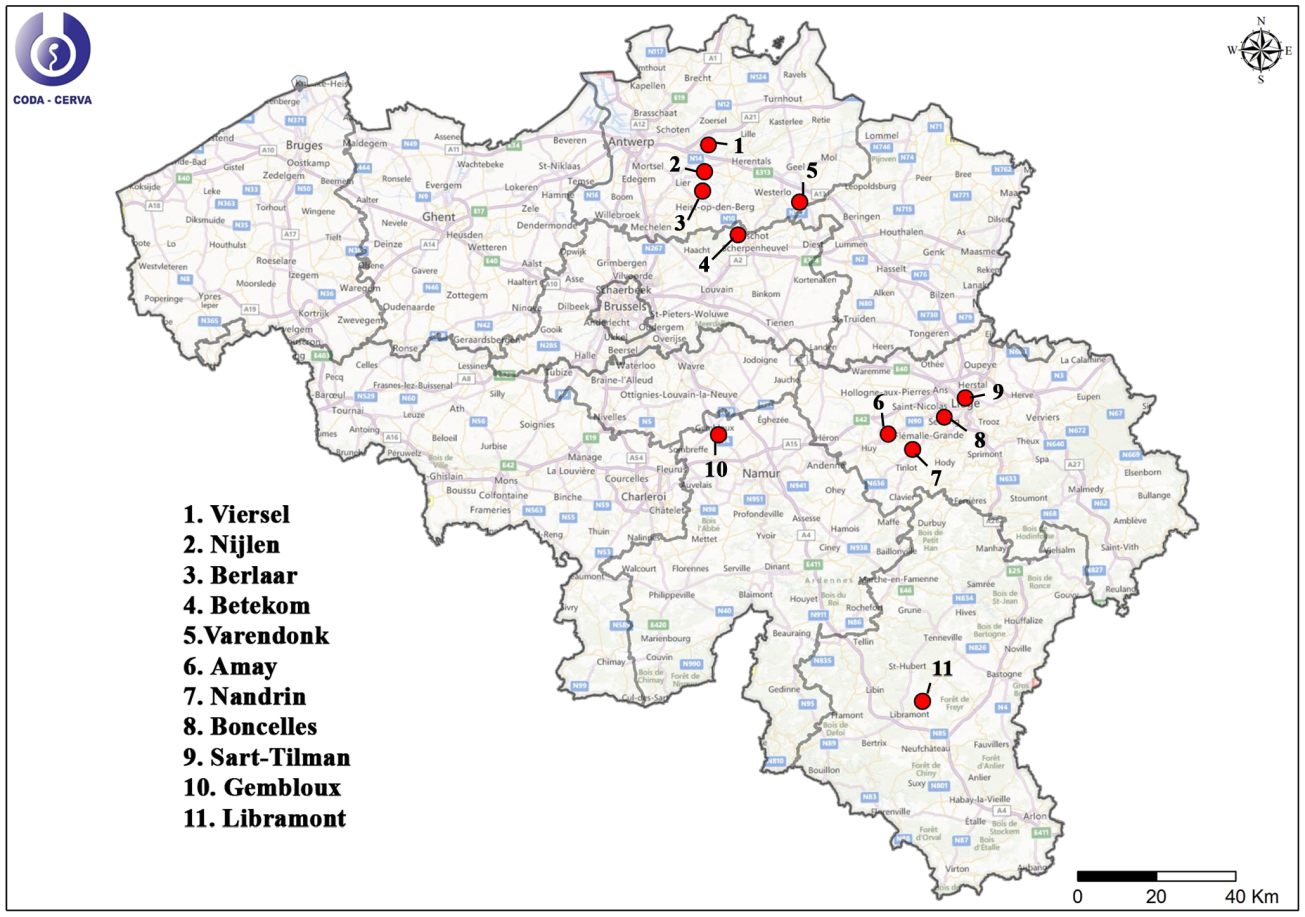
*Culicoides* trapping locations during the *Culicoides* monitoring of 2012.

From May till November 2012, *Culicoides* were caught with Onderstepoort Veterinary Institute (OVI) traps [31] at 12 different locations (Table 1) covering 4 different regions of Belgium (Figure 1): Antwerp (north-east), Liège (east), Gembloux (centre) and Libramont (south). During that time period, *Culicoides* were caught biweekly during one night with the black light traps. Attracted insects were caught in a container containing 60% ethanol. All OVI traps were installed at places where livestock was present in the immediate vicinity (Table 1).

**Table 1:** Detailed information on *Culicoides* trapping locations.

location	livestock	longitude (°E)	latitute (°N)
Antwerp			
Betekom	sheep, deer, chickens	4.79206	51.00200
Varendonk	cows	4.954160	51.085820
Berlaar	cows	4.665713	51.118610
Nijlen	cows	4.693747	51.159744
Viersel	cows	4.63627	51.18454
Liège			
Amay	Chickens, rabbits, sheep	5.185050	50.33694
Boncelles	horses	5.554803	50.567739
Sart-Tilman	cows, sheep, goats,chickens, horses	5.587336	50.576544
Nandrin	horses, cows, pigs	5.358419	50.528464
Gembloux	cows, sheep, pigs	4.72662	50.56509
Libramont	sheep	5.35956	49.92881
	cows	5.35636	49.92931

### Ethics Statement

A permission of access and realization of light trapping of *Culicoides* was given by the farmers of the different sampling locations. No protected species were sampled during this study.

### Morphological Identification, Determination of the Physiological Status and Pool Preparation

The biting midges of each capture place and time were kept separately and were morphologically identified under the stereomicroscope using the key of Delécolle [32] and further stored in 80% ethanol. *Culicoides* from Antwerp, Gembloux and Libramont were identified at subgenus level and female midges belonging to the following subgenera were further examined: *Avaritia* (*C. obsoletus, C. scoticus, C. dewulfi, C. chiopterus), Culicoides (C.pulicaris, C. punctatus, C. impunctatus, C. lupicaris, C. newsteadi, C. deltus, C. grisescens, C. fagineus)* and *Monoculicoides (C. nubeculosus, C. riethi, C. puncticollis)*. Midges belonging to other subgenera were grouped in separate pools designated as ‘other species’. *Culicoides* caught in the region of Liège were identified at species or complex level and females of the following species or complexes were further examined: Obsoletus complex, *C. dewulfi*, *C. chiopterus*, *C. pulicaris* and *C. furcillatus*. Before preparing subgenus, complex or species specific pools of maximum 20 whole *Culicoides*, their physiological status was determined as ‘nulliparous’, ‘parous non-engorged’, ‘blood present’ or ‘blood and eggs present’ as described by Fassotte et al. (2008) [33]. Sixty nine pools were prepared representing 1,359 nulliparous females caught in May in the region of Antwerp (Betekom, Nijlen, Varendonk) and Gembloux, while all other pools only contained parous non-engorged females (904 pools representing 17,461 midges).

### rRT-PCR Analysis of Pools of *Culicoides*

To examine the presence of SBV in *Culicoides*, pools were analyzed by real-time reverse transcription PCR (rRT-PCR) as described before [13]. Briefly, each pool was homogenized in 500 µl Trizol (Life Technologies, Ghent, Belgium) with a 5 mm steel bead (Qiagen, Hilden, Germany) by high speed shaking (3 min, 25 Hz) in a TissueLyser (Qiagen). Following the manufacturer’s instruction, total RNA was extracted from the aqueous phase using the MagMAX Total Nucleic Acid Isolation kit and the MagMAX Express-24 purification system (Life Technologies). RNA was eluted in 90 µl elution buffer. The presence of SBV RNA was analyzed by using the AgPath-ID One Step RT-PCR kit (Life Technologies) following the manufacturer’s instructions in a duplex rRT-PCR for detection of the SBV-S segment [34] and the 18S rRNA from *Culicoides* [35] as an internal control for RNA extraction and amplification. Pools positive for the SBV-S segment were subjected to another rRT-PCR detecting the L segment of the virus [13] (primers and probe sequences were kindly provided by FLI, Germany) using the same one step RT-PCR kit for confirmation. When this second rRT-PCR resulted in data difficult to interpret due to Ct values derived from atypical fluorescence amplification curves, the RNA extract was retested with the same primers but in a two-step PCR with the FastStart TaqMan Probe Master kit (Roche, Basel, Switzerland) following the manufacturer’s instructions after reverse transcription using the M-MLV reverse transcriptase system (Life Technologies, Ghent, Belgium). In the end, only pools that were positive for the S and L segment were considered as SBV positive.

## Results and Discussion

**Figure 2:**
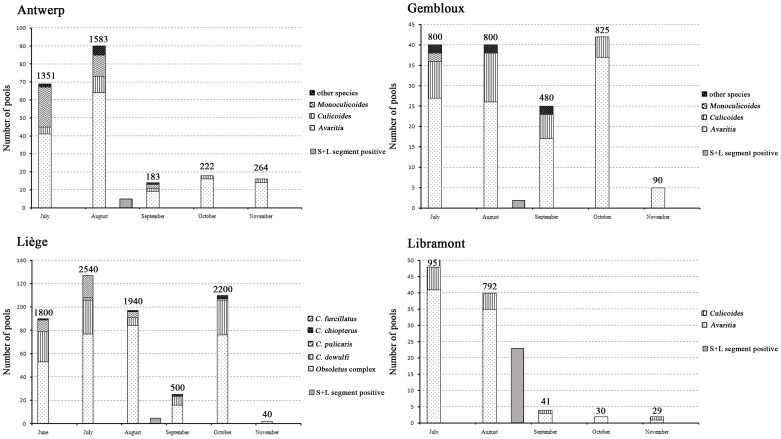
Overview of pools of *Culicoides* examined for the presence of Schmallenberg virus originating from 4 trapping regions in 2012. The numbers mentioned above the bars indicate the exact number of *Culicoides* tested.

The extensive spread of SBV in 2011 in Belgium associated with the induction of high between- and within-herd seroprevalence rates in cattle and sheep [21], [22] raised the question at the beginning of the vector season 2012 if a renewed SBV circulation in hosts and *Culicoides* would be observed. The results of this study show that in all 4 regions where *Culicoides* were sampled, SBV positive pools were found at the beginning of August 2012 (Figure 2), confirming the presence of SBV. Despite the examination of a similar number of pools containing midges caught in July, no SBV positive *Culicoides* could be detected then. These results correlate well with a report describing that SBV was found in a single sheep belonging to a flock held in Namur (Belgium) around mid-July 2012, while most other sheep of the flock became viremic only between mid-August and late September [28]. A limited number of reported SBV positive newborn calves at the beginning of 2013 [27] and a few SBV positive aborted lambs and calves diagnosed at the Belgian reference laboratory CODA-CERVA from November 2012 onwards support an SBV circulation during August 2012.

At Libramont in the south of Belgium, the observed number of positive pools in 2012 was unexpectedly high with 57% (20/35) of *Avaritia* pools and 60% (3/5) of *Culicoides* pools being rRT-PCR positive (Figure 2, Table 2). If only 1 midge per pool was SBV positive, this would corresponds to an infection rate in August 2012 at Libramont of 2.86 and 3.26% in the subgenera *Avaritia* and *Culicoides*, respectively. These percentages are similar to the infection rates of 3.6 and 2.4% reported in Obsoletus complex midges in September 2011 in Antwerp and October 2011 in Liège respectively [13] (unpublished results). The infection rates of 2011 were however reached in the setting of a naïve host population while most potential host individuals were considered seropositive in 2012. It should be taken into account that in the 2011 study, examined pools contained only heads whilst in the present study whole midges were used. Since Ct values for SBV tend to be higher in heads of infected midges than in their abdomen [20], it can be assumed that the true infection rate in 2011 was possibly even higher than the reported values. The most probable explanation for the high infection rate in midges in the south of Belgium is the presence of hosts that had not been infected during the SBV epidemic in 2011. This hypothesis is supported by the reported relatively lower seroprevalence in sheep and cattle in the south of Belgium at the end of 2011 [21], [22] and the presence of a partially seronegative population of wild cervids in southern Belgium at that time [23]. If a fast drop of the acquired immune protection or a non-protective immune response induced by the previous infection would be considered as alternative explanations, it would seem logical that in the other examined regions infection prevalences should be similar to Libramont.

**Table 2:** Detailed overview of Schmallenberg virus positive pools of *Culicoides*.

				Ct value	
location	subgenus/species	collection date	I.C.	S segment	L segment
Antwerp					
Betekom	*Avaritia*	01 Aug 2012	17.07	24.73	26.69
Berlaar	*Avaritia*	03 Aug 2012	13.05	22.01	23.82
Berlaar	*Avaritia*	03 Aug 2012	14.25	25	26.13
Viersel	*Avaritia*	14 Aug 2012	9.84	28.09	31.89
Berlaar	*Avaritia*	14 Aug 2012	10.87	23.67	26.32
Liège					
Nandrin	Obsoletus complex	09 Aug 2012	11.6	27.8	30.06
Nandrin	Obsoletus complex	09 Aug 2012	11.3	34.09	35.18
Boncelles	Obsoletus complex	09 Aug 2012	11.02	30.34	32.38
Amay	Obsoletus complex	22 Aug 2012	11.64	21.6	23.53
Nandrin	Obsoletus complex	22 Aug 2012	12.56	21.68	23.13
Gembloux	*Culicoides*	07 Aug 2012	8.19	30.9	30.81
	*Avaritia*	07 Aug 2012	9.54	31.1	31.51
Libramont	*Culicoides*	08 Aug 2012	15.05	33.96	40.0*
	*Culicoides*	08 Aug 2012	14.98	34.2	38.8*
	*Avaritia*	08 Aug 2012	17.62	34.28	40.0*
	*Avaritia*	08 Aug 2012	17.27	22.12	23.6
	*Avaritia*	08 Aug 2012	17.14	33.46	37.0*
	*Avaritia*	08 Aug 2012	17.22	35.06	40.0*
	*Avaritia*	08 Aug 2012	17.92	35.01	40.0*
	*Avaritia*	08 Aug 2012	16.73	35.84	40.0*
	*Avaritia*	08 Aug 2012	17.91	35.06	40.0*
	*Culicoides*	23 Aug 2012	9.56	33.54	38.76*
	*Avaritia*	23 Aug 2012	10.51	21.71	23.96
	*Avaritia*	23 Aug 2012	10.7	21.02	23.04
	*Avaritia*	23 Aug 2012	10.22	31.94	38.03*
	*Avaritia*	23 Aug 2012	10.3	33.33	37.98*
	*Avaritia*	23 Aug 2012	10.88	21.05	23.64
	*Avaritia*	23 Aug 2012	11.8	30.34	36.35*
	*Avaritia*	23 Aug 2012	10.56	33.24	40.0*
	*Avaritia*	23 Aug 2012	10.93	20.99	22.26
	*Avaritia*	23 Aug 2012	11.31	21.54	23.89
	*Avaritia*	23 Aug 2012	10.86	19.35	21.0
	*Avaritia*	23 Aug 2012	10.36	33.07	38.79*
	*Avaritia*	23 Aug 2012	10.32	33.22	40.0*
	*Avaritia*	23 Aug 2012	10.92	19.91	21.53

* tested in two-step rRT-PCR.

I.C. = internal control.

However, the infection prevalences in August in the other regions were however clearly lower (0.4, 0.3, and 0.2% in *Avaritia* in Antwerp, Liège and Gembloux, respectively, and 0.4% in *Culicoides* in Gembloux). In those regions, non-protected sheep and calves borne after the 2011 epidemic have probably allowed the replication of the virus and served as a source for SBV infection of the midges.

The sudden appearance of SBV infected midges in all studied regions in August raises the question as to where the virus came from. A first possibility could be that the virus had overwintered in either its host or its vector. Since only a short viraemia occurs in sheep and cattle [1], [24], [25], [36], [37], it seems unlikely that the virus overwintered in these animals. Overwintering in another, so far unidentified, animal host could be another possibility. When overwintering in *Culicoides* is considered, this could either occur in the low number of adult midges that are capable to survive Belgian winter conditions [38] or in infected eggs or larval stages after transovarial virus transmission. To get a first idea if overwintering in the vector had occurred by transovarial virus transmission in Belgium, 69 pools representing 1,359 nulliparous females caught in May 2012 at places where SBV had circulated in 2011 were tested (Table 3). The fact that all were found negative provides an indication that transovarial transmission is not likely to occur. This should, however, be further investigated since it was recently reported that SBV RNA was detected in midges considered as nulliparous based on visual inspection in Poland in 2012 [18]. Laboratory based studies will probably be necessary to unambiguously show whether transovarial transmission in biting midges occurs or not. Another possibility that cannot be excluded at the moment is that, besides *Culicoides*, other hematophagous insects can function as (overwintering) vectors for SBV. In this respect, no SBV has been found in 868 hibernating mosquitoes in the Netherlands collected from January to March 2012 [39]. Although no SBV positive insect vectors have been detected during winter yet, the finding that sheep became viremic for SBV in the beginning of January 2013 in Germany during a period characterized by a rise of the minimum temperature above 5°C supports the hypothesis that SBV overwinters in hematophagous insects [40]. Alternatively to overwintering in hosts or vectors, the observed presence of SBV in 2012 in Belgium could also have been the result of a reintroduction of the virus by arthropods coming in from neighbouring countries since SBV circulation in 2012 has been reported in France, Germany and The Netherlands [16], [26], [29].

**Table 3:** Schmallenberg virus detection in nulliparous female midges caught in May 2012.

	Antwerp	Gembloux	# SBV pos
*Avaritia*	24 (476)	31 (620)	0
*Culicoides*	5 (85)	–	0
*Monoculicoides*	8 (160)	–	0
Other species	1 (18)	–	0
Total	38 (739)	31 (620)	0

Similar to the sudden appearance of SBV in *Culicoides* in August 2012, the abrupt absence of SBV in examined pools of *Culicoides* caught in September 2012 and later is remarkable (Figure 2). Based on the knowledge of the fast spread of SBV during the 2011 epidemic, this is most probably caused by the rapid infection of all residual non-protected animals, associated with the induction of a protective immune response that prevented further spread.

**Figure 3:**
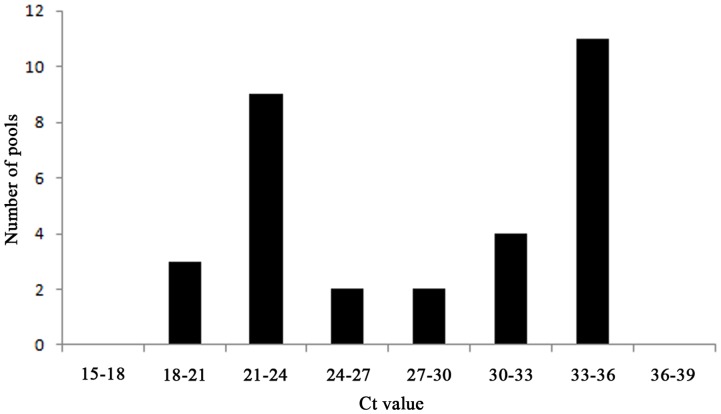
Ct value frequency distribution of Schmallenberg virus positive pools of *Avaritia*.

Due to the outbreak of bluetongue virus in 2006 in Central and Northern Europe [41] and the recent SBV emergence, scientists have been confronted with two viruses that were immediately suspected to be spread by biting midges once first diagnosis in animal hosts had occurred. In the urge to identify responsible vector species as fast as possible, several *Culicoide*s species have been proposed as putative vectors based on the identification of the virus in pools of field caught *Culicoides* by rRT-PCR [14], [17]–[19], [35], [42]–[44]. Several aspects related to (1) the diagnostic technique, (2) arbovirus replication and dissemination characteristics in their vectors and (3) pool composition, however, cause that such results should be interpreted with caution, particularly if only a limited number of positive pools are found [20], [45]. Two main obstacles are that i) rRT-PCR also detects non-infectious viral RNA, making it uncertain that obtained Ct values truly represent virus capable of infecting new hosts, and ii) rRT-PCR positive whole midges do not necessarily represent vectors producing transmissible virus since barriers preventing virus to enter or escape from the midgut and preventing virus dissemination through the haemocoel exist in *Culicoides* that may prevent dissemination of the virus to the head and salivary glands after uptake of an infectious blood meal [46], [47]. Upon the emergence of SBV, these issues were partially addressed by some studies that attempted detecting SBV in species-specific pools consisting only of *Culicoides’* heads [13], [15] since this implicates that rRT-PCR positive pools contain most probably fully disseminated infections. However, this method is labor intensive and therefore offers only chance to detect virus positive *Culicoides* in the restricted number of pools tested when vector competence for the studied virus is high [20]. Recently, a new approach to evaluate arbovirus dissemination via rRT-PCR in field caught whole *Culicoides* was proposed based on the results obtained with laboratory grown *C. sonorensis* midges orally fed with SBV or BTV-infected blood via a membrane system [20], [30]. It was found that SBV infection of a susceptible vector species resulted in a bimodal distribution of the obtained Ct values. Ct values close to the first peak indicate a fully disseminated infection and presence of transmissible virus while Ct values close to the second peak indicate sub-transmissible infections. SBV infection of refractory species (*C. nubeculosus*) did not result in a bimodal distribution of obtained Ct values. When a similar data analysis was applied to the Ct values obtained in this study for the 31 SBV-S segment positive pools of *Avaritia* (Table 2), a bimodal distribution could be found with a first peak of Ct values between 21 and 24 and a second peak with Ct values between 33 and 36 (Figure 3). Following the interpretation of the proposed approach [20], this result predicts that the subgenus of *Avaritia* contains competent SBV vector species. This is in line with previous publications suggesting Obsoletus complex, *C. chiopterus* and *C. dewulfi* midges as putative vectors based on rRT-PCR analysis of species-specific pools of heads [13], [15] and therefore represents a field validation of the proposed approach.

The necessity for a relatively high number of rRT-PCR positive midges of a certain species to reliably estimate if a bimodal distribution is present will probably sometimes limit the applicability of this approach to assess the vectorial competence of field caught midges. In the present study, 4 SBV-S positive pools containing midges of the subgenus *Culicoides* were found with Ct values for the SBV-S segment between 30.9 and 34.2 (Table 2). This number is clearly not sufficient to assess if species belonging to the subgenus *Culicoides* are refractory species to SBV or if they (or a specific species of the subgenus *Culicoides*) are susceptible and the pool contained individuals with sub-transmissible amounts of SBV. Similar inconclusive results were obtained by rRT-PCR analysis of pools of heads of *C. pulicaris* since these were positive for the S segment of SBV but not the L segment [13]. It seems advisable to interpret these data in a conservative way and not to propose *C. pulicaris* as a competent vector at this moment.
